# Retinitis pigmentosa and ocular blood flow

**DOI:** 10.1186/1878-5085-3-17

**Published:** 2012-12-03

**Authors:** Katarzyna Konieczka, Andreas J Flammer, Margarita Todorova, Peter Meyer, Josef Flammer

**Affiliations:** 1 Department of Ophthalmology, University of Basel, Mittlere Strasse 91, Basel, CH-4031, Switzerland; 2 Cardiovascular Center, Cardiology, University Hospital Zurich, Zurich, CH-8091, Switzerland

**Keywords:** Retinitis pigmentosa, Primary vascular dysregulation syndrome, Ocular blood flow, Endothelin, Integrative medicine, Predictive, preventive and personalised medicine

## Abstract

Is the concept of integrative, preventive and personalised medicine applicable to the relationship between retinitis pigmentosa (RP) and ocular blood flow (OBF)? RP encompasses a group of hereditary diseases of the posterior segment of the eye characterised by degeneration, atrophy and finally loss of photoreceptors and retinal pigment epithelium, leading to progressive visual loss. Many different mutations affecting different genes can lead to the clinical picture of RP. Even though the disease has a clear genetic background, there are obviously other factors influencing the manifestation and progression of RP. In this review, we focus on the role of OBF. There is evidence that, in PR patients, OBF is more reduced than one would expect secondary to the retinal atrophy. The main cause of this additional component seems to be primary vascular dysregulation (PVD) syndrome. As PVD syndrome is partly treatable, a vascular evaluation of RP patients is meaningful. Based on the outcome, a targeted individualised, preventive or supportive treatment might be introduced in selected RP patients.

## Review

### Introduction

**Figure 1:**
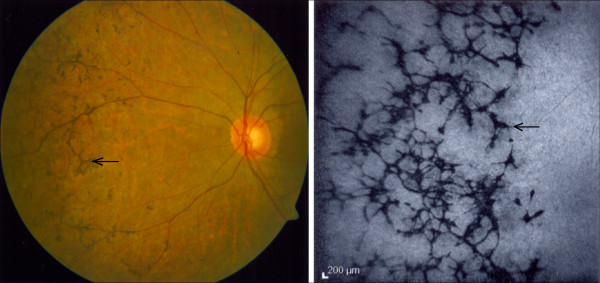
**Fundus photograph of a patient with retinitis pigmentosa with the typical bone-spicule pigmentary changes (*arrows*). ***Left*: photograph; *right*: fluorescein angiogram.

Retinitis pigmentosa (RP) encompasses a large group of hereditary diseases of the posterior segment of the eye characterised by degeneration, atrophy and finally loss of photoreceptors and retinal pigment epithelium (RPE), leading to progressive visual loss. The term ‘retinitis’ refers to an inflammatory component. Indeed, most dystrophic and degenerative diseases are accompanied by low-grade inflammation. The term ‘pigmentosa’ refers to the pigmentary changes with a perivascular ‘bone-spicule’ configuration in the fundus of the eye (Figure 1).

The fact that a number of different mutations affecting different genes [1] lead to the clinical picture described phenomenologically as RP explains the heterogeneity of the phenotypes, including age of onset, rate of progression and severity of the disease. Even though the disease has a genetic background, we assume that additional factors influence the manifestation of the disease. One potential modifying factor is disturbed ocular blood flow (OBF). Indeed, reduced OBF in RP patients has been described [2-4]. Blood flow is more or less always reduced in atrophic tissue, secondary to a decreased demand for supply. However, in this review, we summarise and focus on findings indicating an additional primary component of OBF reduction and explore the potential causes of such a primary component. Finally, we discuss the potential benefit of a vascular evaluation of RP patients with regard to prophylactic and supportive treatment in selected patients.

### Phenomenology of RP

**Figure 2:**
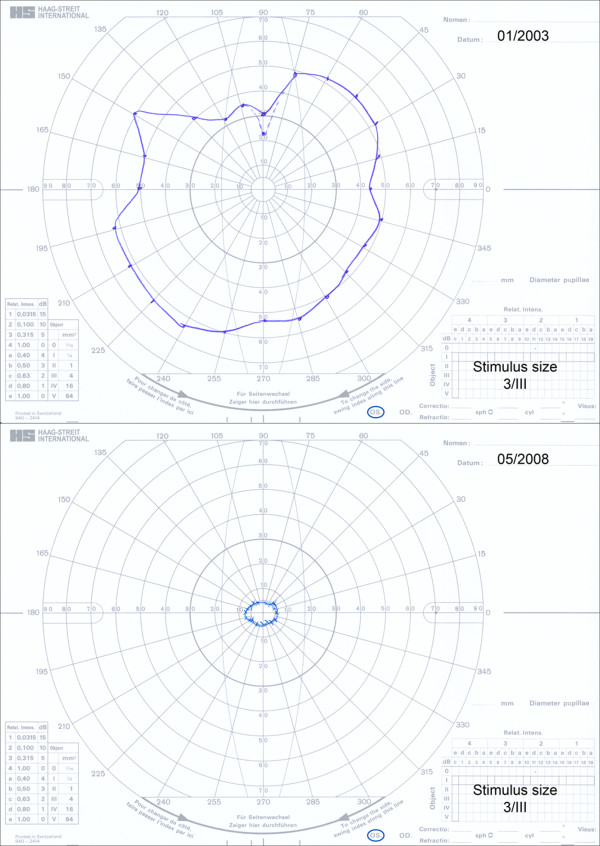
**Goldmann visual fields of a RP patient demonstrating progressive concentric constriction. ***Top*: mid-stage RP; *bottom*: late-stage RP.

**Figure 3:**
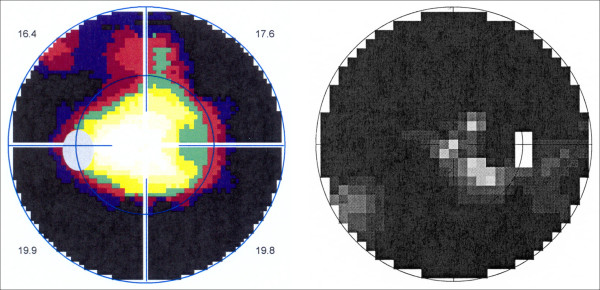
**Octopus perimetry demonstrating the extensive visual field loss. ***Left*: mid-stage RP; *right*: late-stage RP.

**Figure 4:**
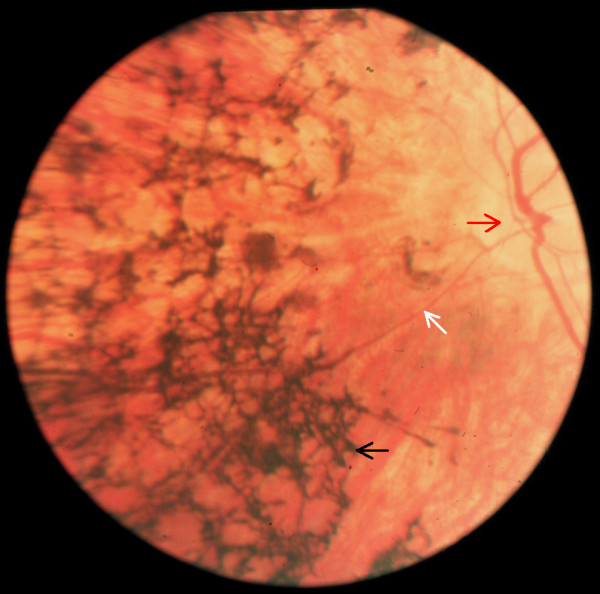
**Fundus photograph in patient with late-stage RP with the classic triad.** Bone-spicule retinal pigmentation (*black arrow*), retinal vessel attenuation (*white arrow*) and waxy disc pallor (*red arrow*).

**Figure 5:**
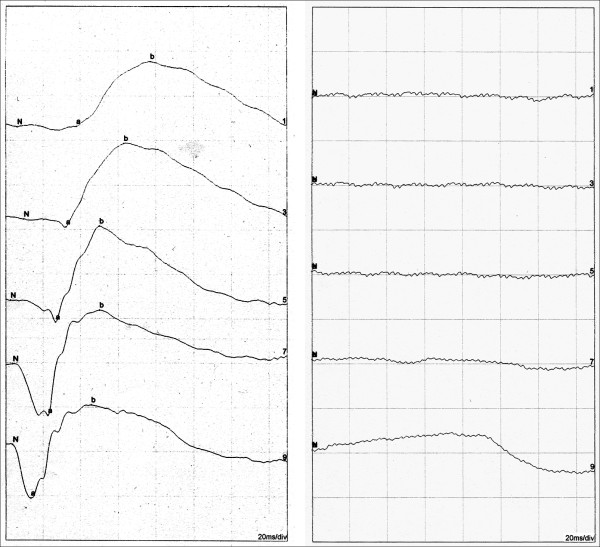
**Scotopic electroretinography (ERG), ISCEV standard (International Society for Clinical Electrophysiology of Vision).** Normal ERG responses in healthy subject (*left*) and extinguished responses in RP patient (*right*).

**Figure 6:**
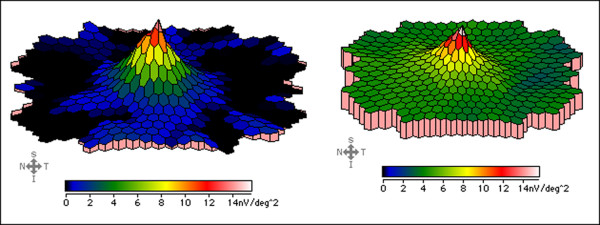
**Multifocal electroretinogram.** Severely attenuated paracentral responses in RP patient (*left*) in comparison to normal responses in a healthy subject (*right*).

**Figure 7:**
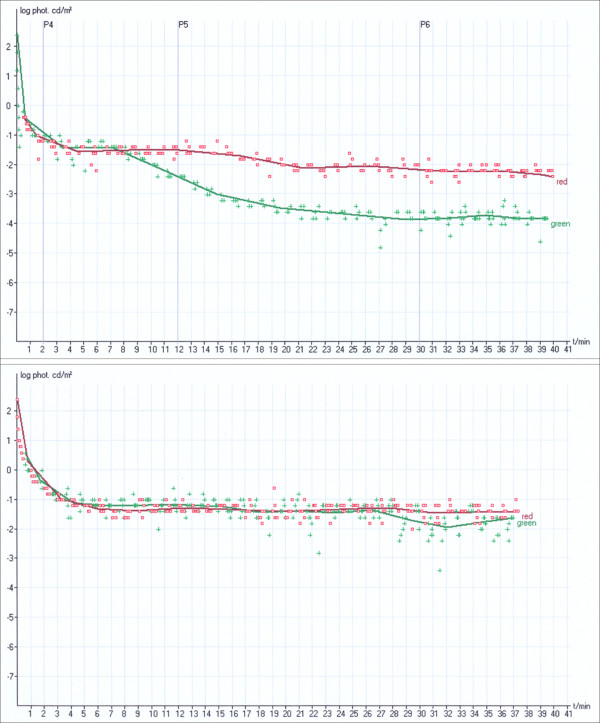
**Dark adaptation.** Dark adaptation of rods (*green line*) and cones (*red line*) in a healthy subject (*top*) and disturbed dark adaptation (*loss of red-green dissociation*) in RP patient (*bottom*).

As mentioned above, RP refers to a group of diseases of progressive retinal degenerations characterised by loss of photoreceptors and RPE with corresponding loss of function. The disease has a hereditary basis. The prevalence of RP is estimated to be one case among 3,000–5,000 individuals, with a total of about two million affected persons worldwide [5]. The disease starts typically (but not always) with night blindness followed by bilateral, symmetric, progressive concentric constriction of the visual field (Figures 2 and 3), finally leading to tunnel vision or even complete blindness. By fundoscopy, the ophthalmologist finds a bone-spicule retinal pigmentation starting in the periphery and extending slowly towards the centre of the retina, attenuated retinal vessels and, in late stages, a waxy-pale optic nerve head (Figure 4). The electroretinography reveals reduced rod and cone responses (Figures 5 and 6), and dark adaptation is reduced and finally lacking (Figure 7).

**Figure 8:**
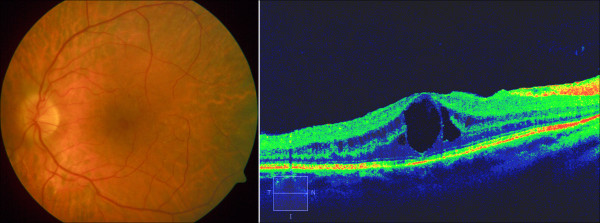
**Cystoid macular oedema in RP patient. ***Left*: fundus photograph; *right*: optical coherence tomography image.

In addition, RP can be accompanied by cataract [6], open-angle glaucoma, refractive errors [7], keratoconus, optic nerve head drusen and cystoid macular oedema [8] (Figure 8).

It should be noted that both the types and the severity of clinical symptoms and signs, as well as age of onset and rate of progression, vary markedly among patients (reviewed in [9]). Symptoms may already start in childhood; however, they more often begin in early adulthood and sometimes in mid-adulthood. Although the progression of the disease is variable, typically, severe visual impairment occurs most often by the age of about 40–50 years [1].

This large clinical heterogeneity is at least partly explained by the genetic heterogeneity. However, after a short summary of the genetics of RP, we will focus our discussion here mainly on the question of whether disturbed blood flow may also contribute.

### Genetics of RP

The condition can be inherited in an autosomal-dominant, autosomal-recessive or X-linked fashion. Non-Mendelian inheritance patterns, such as digenic [10] and maternal (mitochondrial) [11] inheritance, have also been reported. For the genetics of RP, we refer to a recently published review [1].

**Figure 9:**
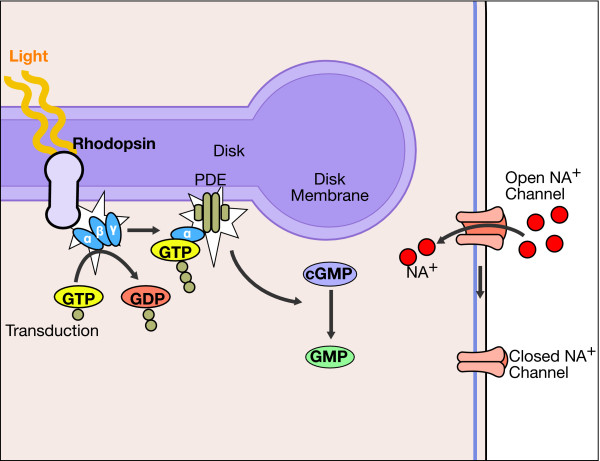
**Phototransduction in rod photoreceptors.** Mutations of genes involved in this process can lead to RP (Adapted from [14] with permission).

**Figure 10:**
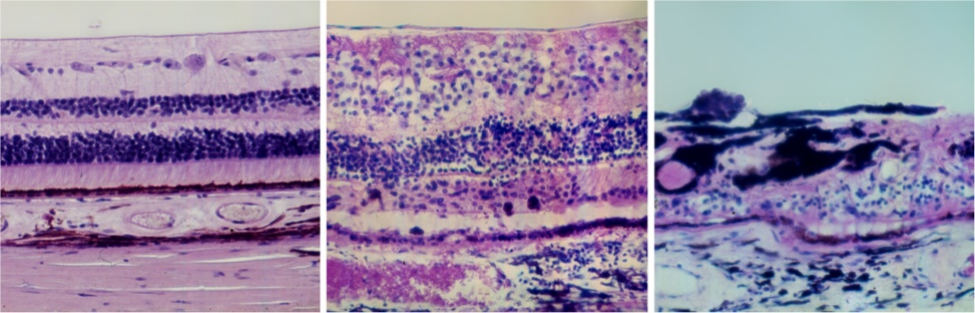
**Histological section through the human retina. ***Left*: healthy retina; *middle*: retina of a patient with mid-stage RP; *right*: retina of a patient with late-stage RP.

However, the feature shared by all these types of mutations is that they occur in genes coding for proteins involved in vision, either at the level of rods and cones or at that of the pigment epithelium (Figure 9). In the course of the disease, the photoreceptors undergo apoptosis [12], which results in reduced outer nuclear layer thickness of the retina (Figure 10). The pigment deposits, also described as bone-spicule pigmentation, result from both RPE degeneration and RPE migration into the neural retina in response to photoreceptor cells' death [13] (Figure 1).

The exact mechanism of cell death in RP is not yet known. There are indications, however, that oxidative stress is also involved [15]. Any condition that increases or decreases oxidative stress in the retina is therefore of interest.

### RP and ocular blood flow

It is meanwhile well established that OBF in RP patients is reduced not only in the retina [3] and choroid [4], but also in the retroocular vessels [2]. The question arises whether this is only secondary to the retinal atrophy or whether there is a primary component of OBF dysfunction (such as an atrophy-independent reduction of OBF) potentially contributing to the damage. In this context, two questions are important: (1) At what stage of the disease does the onset of OBF reduction occur? (2) Is blood flow reduction confined to the eye?

**Figure 11:**
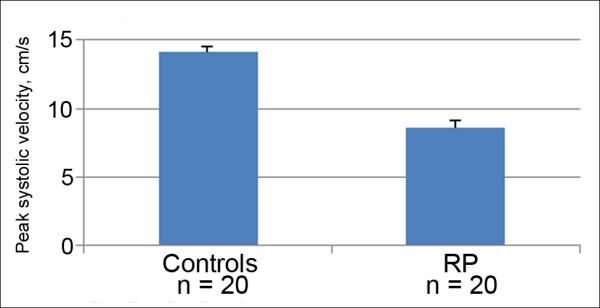
**Peak systolic velocities (mean ± SEM) in posterior ciliary arteries.** Control subjects (*left*) and patients with early-stage RP (*right*) (based on [2]).

Interestingly, reduced blood flow velocity in the retina (increase of the arteriovenous passage time) has been observed already in the early stages of RP before the appearance of any ophthalmoscopic signs [16]. With colour Doppler imaging, Cellini et al. also demonstrated reduced peak systolic velocities in both ophthalmic arteries and posterior ciliary arteries (Figure 11) (not explained by the occurrence of other general disorders) [2]. Furthermore, Cellini et al. also demonstrated that blood flow disturbance in RP patients was not confined to the eye, but also occurred in the periphery. With laser Doppler flowmetry, they demonstrated reduced baseline peak flow in the cutaneous capillary of the finger and significantly longer recovery time after cold provocation [2]. Furthermore, these patients had increased endothelin-1 (ET-1) plasma levels [2], and ET-1 plasma level correlated with the decrease of peak systolic velocity both in the ophthalmic artery and in the posterior ciliary arteries [2]. Again, this was observed in RP patients in the early stages of the disease.

Such an increase in ET-1 plasma levels in RP patients has been described repeatedly [2,17,18] but not confirmed by all investigators [19].

**Figure 12:**
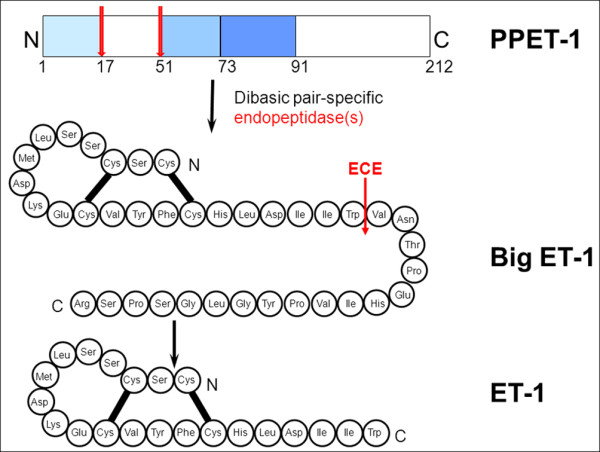
**Endothelin.** ET-1 arises by cleaving the precursor molecules (Adapted from [14] with permission).

**Figure 13:**
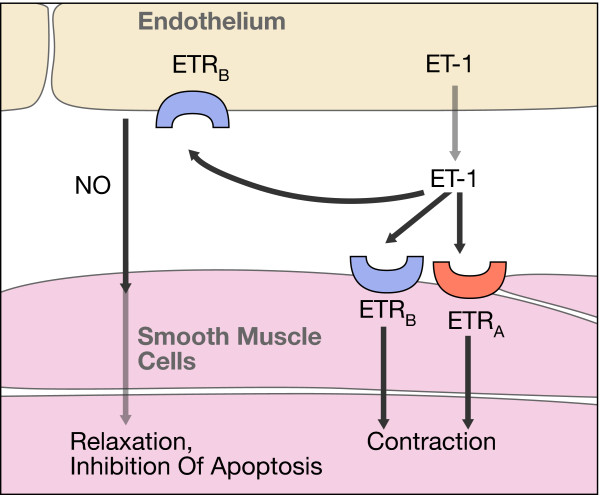
**Endothelin receptors.** In humans, the effects of ET-1 are mediated by two types of ET receptors: the type-A receptor (ET_A_) and the type-B receptor (ET_B_) (Adapted from [14] with permission).

It is at present not known why ET-1 levels are increased at least in some RP patients. Endothelin (ET) (Figures 12 and 13), identified in 1988 by Yanagisawa and colleagues [20], is a strong endogenous vasoconstrictor. Endothelins are a family of 21 amino acid peptides, where the predominant isoform is ET-1. ET-1 is essentially produced by vascular endothelial cells. However, more or less, any cell can produce ET-1 if the cell is under stress. Particularly important is hypoxic stress, which leads to an increase of HIF-1 alpha and thereby increased expression of ET-1. Therefore, the observed increase of plasma level could be secondary to hypoxic or oxidative stress somewhere in the body. It is at present unknown whether the ET-1 level in the eye is even more increased than in the plasma. It is known that ET-1 can be synthesised and secreted in RPE cells [21]. This locally secreted ET-1 may also play a role in cell proliferation, migration and repair processes in the retina. Independent of the cause of the ET-1 increase in the plasma, this increase reduces OBF, particularly in the choroid and the optic nerve head [22] and, in high concentrations, even in the retina [23].

### The impact of primary vascular dysregulation syndrome in RP

We hypothesise that primary vascular dysregulation (PVD) syndrome [24-26] might be a main cause for both the observed findings in blood flow reduction and the increase in ET-1.

PVD is a predisposition to react differently to a number of stimuli like coldness [24,25] and physical or emotional stress. The most prominent sign of PVD is the dysregulation of vessels, hence the name of the syndrome [27]. The most notable sign in the vessels are vasospasms. This explains why for PVD the term vasospastic syndrome [28] was used in the past.

**Figure 14:**
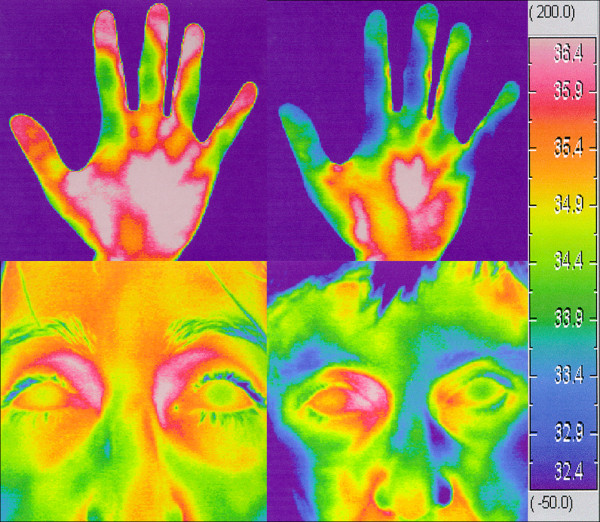
**Thermography of hands and faces.** A subject without PVD (*left*) and a subject with PVD (*right*) (Adapted from [32] with permission).

**Figure 15:**
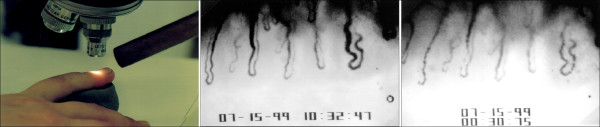
**The nailfold capillary microscopy examination. ***Left*: examination setting with cooling device; *middle*: picture from a video with normal capillary blood flow; *right*: picture from the video demonstrating blood flow cessation after cold provocation in a PVD subject (Adapted from [32] with permission).

**Figure 16:**
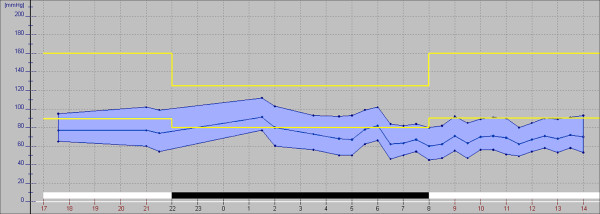
**Outcome of blood pressure monitoring. ***Yellow*: normal range; *blue*: systolic, diastolic and mean blood pressure of a RP patient.

The main signs and symptoms of PVD are cold hands and/or feet [25] (Figure 14), particularly when exposed to cold (Figure 15) or emotional stress and low blood pressure [29]. Typically, these subjects have mildly increased ET-1 plasma levels [30]. One cause of the systemic hypotension (Figure 16) observed in these subjects is the reduction of the reabsorption of sodium in the proximal tubule of the kidneys [31]. This, in turn, is due to the increase of prostaglandin E2 (PGE2) by the ET-1. PVD subjects also often have reduced feelings of thirst [30], which is also due to the increased ET-1 plasma levels.

Furthermore, they respond very sensitively to certain drugs—most probably due to altered expression of ABC-transport proteins [33]. PVD subjects also require a longer time to fall asleep, especially when they are cold [34] (as warm feet are generally a prerequisite for falling asleep). They also suffer more often from headaches and migraines [35] and have increased pain sensation [36] (ET level influences the threshold of pain sensation). PVD occurs more often in females [37] and in thin subjects [37-39].

**Figure 17:**
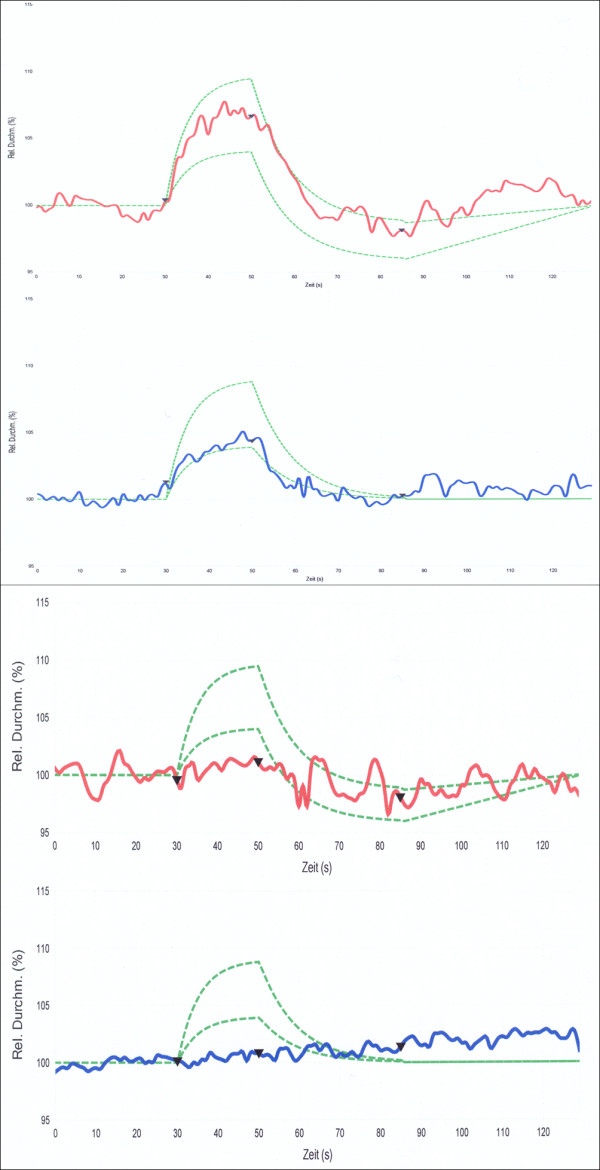
**Vascular response to light measured with a Retinal Vessel Analyser. ***Top*: normal responses of both arteries and veins; *bottom*: reduced responses of both arteries and veins in a RP patient. *Red lines*: arteries; *blue lines*: veins.

Interestingly, most of these signs and symptoms typical of PVD subjects also seem to occur more frequently in RP patients than in unselected controls (Konieczka K et al., unpublished work). We therefore discuss here the link between PVD and OBF. PVD syndrome also includes disturbed autoregulation of ocular perfusion [40], spatial irregularity of retinal vessels and reduced neurovascular coupling [41] (Figure 17). Disturbed regulation of OBF leads to unstable blood flow (and therefore unstable blood supply), and this, in turn, leads to an increase in free radicals and chronic oxidative stress. Indeed, there are many indications that oxidative stress is increased in PVD [42].

The high prevalence of PVD in RP patients might explain the primary component of OBF dysfunction in RP patients.

### Therapeutic consequences

Dealing with a severe disease, any treatment ameliorating this condition would be welcome. On the other hand, it would be wise to avoid encouraging false hope. We know from clinical experience that the symptoms of PVD can be reduced to some extent. If blood pressure is very low, increased salt intake increases blood pressure slightly but improves vascular regulation markedly. The same observation has been made with fludrocortisone [43]. The other symptoms of dysregulation can be mitigated either by magnesium [44] or by a low dose of calcium channel blockers (CCBs) [45] or even omega-3 fatty acids [46,47].

Interestingly, it has been shown that central visual field defects progress slower in RP patients treated with the CCB nilvadipine [48]. Furthermore, in a patient with the clinical picture of RP, without a genetic history but with chronic hypomagnesaemia, the visual field progression was stopped after magnesium (a physiological CCB) substitution [49]. Magnesium and CCBs both have some neuroprotective effect and improve blood flow regulation. They reduce the vasoconstrictive effect of ET-1 [50,51].

Interestingly, diet rich in omega-3 fatty acids—which we often use to treat PVD—can also slow mean annual rates of decline of visual acuity in RP patients receiving vitamin A [52]. Furthermore, antioxidants (used to treat PVD subjects) may also be helpful for RP patients. Indeed, oxidative damage is under investigation as a possible therapeutic target in RP disease [15,53].

As OBF dysfunction, potentially due to PVD syndrome, occurs often in RP patients, we consider the vascular evaluation of such patients meaningful. If reduced OBF in the context of PVD syndrome is diagnosed, a supportive treatment with a diet rich in both omega-3 fatty acids and antioxidants and with magnesium (or, in selected patients, even with low-dose CCBs) might be helpful. If blood pressure is very low, increased salt intake or, in extreme cases, even low-dose fludrocortisone might be worthwhile. We would like to emphasise, however, that such recommendations are mainly based on clinical experience. Scientific studies are unfortunately, at present, still sparse.

## Conclusions

RP is a disease with a clear genetic background. However, OBF is more reduced in RP than one would expect secondary to the retinal atrophy. The main cause of this OBF reduction seems to be PVD syndrome. As PVD syndrome is partly treatable, an individualised treatment [54,55] might be introduced in selected RP patients based on the outcome of a vascular evaluation.

Further studies are recommended to establish the relationship between reduced OBF and the different stages of the RP disease and to confirm our hypothesis that the main cause of this blood flow reduction is the PVD syndrome. Furthermore, a more detailed relationship between the vascular dysfunction and other involved factors such as ET or nitric oxide should be established. Controlled studies evaluating the effect of vascular treatment on visual function will be crucial. While waiting for the outcomes of such studies, we recommend simple, not expensive treatments such as magnesium or omega-3 fatty acids in targeted selective patients [54,55].

## Abbreviations

CCB: Calcium channel blocker; ET: Endothelin; ET-1: Endothelin-1; OBF: Ocular blood flow; PVD: Primary vascular dysregulation; RP: Retinitis pigmentosa; RPE: Retinal pigment epithelium.

## Competing interests

The authors declare that they have no competing interests.

## Authors’ contributions

All authors have contributed to the manuscript. All authors read and approved the final manuscript.
